# Sugar-terminated carbon-nanodots stimulate osmolyte accumulation and ROS detoxification for the alleviation of salinity stress in *Vigna radiata*

**DOI:** 10.1038/s41598-022-22241-w

**Published:** 2022-10-20

**Authors:** Mahima Misti Sarkar, Nibedita Pradhan, Rewaj Subba, Puja Saha, Swarnendu Roy

**Affiliations:** 1 Plant Biochemistry Laboratory, Department of Botany, University of North Bengal, Raja Rammohunpur, Dist. Darjeeling, West Bengal 734013 India; 2 School of Bioscience, Indian Institute of Technology, Kharagpur, West Midnapore, West Bengal 721101 India; 3 Microbiology Laboratory, Department of Botany, University of North Bengal, Raja Rammohunpur, Dist. Darjeeling, West Bengal 734013 India

**Keywords:** Plant sciences, Nanoscience and technology

## Abstract

In recent times, nanotechnology has emerged as an efficient tool to manage the adverse effect of environmental stresses on plants. In this connection, carbon-nanodots (CNDs) have been reported to ameliorate the negative impacts of salinity stress. Further, surface modification of CNDs is believed to augment their stress-alleviating potential, however, very little has been known about the potential of surface-functionalized CNDs. In this purview, two sugar (trehalose and glucose) terminated CNDs (CNPT and CNPG) have been synthesized and assessed for their stress-alleviating effects on *Vigna radiata* (a salt-sensitive legume) seedlings subjected to different concentrations of NaCl (0, 50, and 100 mM). The synthesized CNDs (CNPT and CNPG) exhibited a hydrodynamic size of 20–40 nm and zeta potential of up to − 22 mV with a 5–10 nm core. These water-soluble nanomaterials exhibited characteristic fluorescence emission properties viz*.* orange and greenish-yellow for CNPT and CNPG respectively. The successful functionalization of the sugar molecules on the CND cores was further confirmed using FTIR, XRD, and AFM. The results indicated that the application of both the CNDs improved seed germination, growth, pigment content, ionic and osmotic balance, and most importantly, the antioxidant defense which decreased ROS accumulation. At the same time, CNPT and CNPG exhibited no toxicity in the *Allium cepa* root tip bioassay. Therefore, it can be concluded that sugar-terminated CNDs improved the plant responses to salinity stress by facilitating sugar uptake to the aerial part of the seedlings.

## Introduction

Recent studies have revealed the role of sugar molecules in plant growth and development along with defense mechanisms^1–3^. Glucose, sucrose, and trehalose-6-phosphate are the most important sugar molecules that are involved in the regulation of various growth and metabolic processes while acting as signaling molecules independently of the basal functions^1^. Sugar molecules and sugar alcohols are low molecular weight compounds that also function as organic osmolytes^4^. Being an osmolyte, sugars not only function as osmoprotectants but also modulate the function of genes for improved stress tolerance^5^. Moreover, the sugars are involved in facilitating many physiological processes viz*.* seed germination, photosynthesis, flowering, senescence, and also in signaling cascade and transcriptional control to combat the stress-induced impacts^6^. In this connection, the exogenous application of sugars has been reported to promote seed germination and flowering, increase photosynthesis, and delay senescence under stressed conditions^7^.

Among the abiotic stresses, salinity imposes a drastic impact on plant growth and development. Crop plants are highly affected by salinity due to their salt sensitivity and thus crop production is estimated to be reduced by 50% of the total yield^8^. Salinity stress mainly imparts a brutal effect on plants by increasing osmotic imbalance, ion (Na^+^) toxicity, and overaccumulation of reactive oxygen species (ROS)^9^. External application of sugars, such as trehalose, glucose, and sucrose has been reported to reduce the adverse effect of salinity by increasing growth, photosynthesis, osmotic balance, membrane stability, antioxidant defense, and decreasing ROS accumulation in plants^10–12^. However, the uptake of sugar molecules by plant roots is limited by the availability of transporters and dependency on active transportation^13^. Therefore, devising strategies for improving the transportation of sugar molecules in the plant system will be beneficial for imparting better growth and stress tolerance.

In this context, nanoparticles have been known to be effectively used as carrier molecules for augmenting the transportation of beneficial molecules viz*.* agrochemicals including pesticides, fertilizers, and nutrient molecules to confer good health and protection to crops^14^. Moreover, several nanomaterials can facilitate species-independent passive delivery of several bioactive molecules into the plant^15^. Carbon nanomaterials are one of the most promising nanomaterials with an increasing number of articles claiming its potential role as a growth inducer, herbicide, and pesticide^16^. In this connection, CNDs have exhibited immense potential in the improvement of growth and yield in several crop plants^17,18^. The CNDs are also observed to improve the photosynthetic rate in both C3 and C4 plants up on foliar application, which ultimately improved the growth of the plants^19^. Moreover, CNDs are one of the most impactful nanoparticles having great potential that can be used for the alleviation of salinity stress^20^. In this connection, CNDs can effectively reduce the detrimental effects of salinity by improving germination, growth, chlorophyll content, crop yield, and quality^21,22^. CNDs are also observed to improve the enzymatic and non-enzymatic antioxidant properties of plants under salinity stress^23^. Moreover, functionalized CNDs have been observed to impart greater efficacy by improving chlorophyll content, osmolyte content, antioxidant enzyme activity, membrane stability index, and reducing MDA, and H_2_O_2_ contents in plants under salinity stress^24^. For example, putrescine-functionalized CNDs have been observed to improve the growth, photosynthesis, and antioxidant activities of saline-stressed plants^25^.

The application of surface functionalization of nanoparticles for the alleviation of the negative impact of abiotic stresses is gaining interest. Therefore, in this study, the potential of CNDs terminated with two osmolyte sugar molecules—glucose and trehalose were assessed for the alleviation of salinity stress in *Vigna radiata* seedlings. In this context, the stress-alleviating role of the sugar-functionalized CNDs was studied in terms of germination and post-germination parameters including growth, chlorophyll, osmolyte content, ion accumulation, antioxidant defense, and ROS accumulation. This study for the first time reports the beneficial role of sugar-terminated CNDs (CNPT and CNPG) in the alleviation of salinity stress in plants.

## Material and methods

### Synthesis of trehalose and glucose-terminated CNDs

Carbon-nanodots were prepared from two sugars (glucose and trehalose) using the previously reported method of Bhunia et al.^26^ and Pradhan et al.^27^. Briefly, 33 mg/mL aqueous solution of glucose/trehalose was prepared and to 3 mL of this sugar solution, 10 µL of concentrated HCl was added. The acidic sugar solution was then heated at ~ 90–100 °C for 2 h while the colourless solution gradually becomes deep brown with heating. The complete carbonization of sugar was further tested by adding a drop of nanoparticle solution to a large volume of acetone. The unreacted sugar appeared as a white precipitate. Next, the reaction was stopped by lowering the temperature to room temperature and adding sodium hydroxide solution to adjust the solution pH to 7.4. The nanoparticle solution was purified by overnight dialysis using a dialysis membrane of MWCO 12 KDa. To calculate the weight per cent (~ 60%), a part of the nanoparticle solution was dried and then weighed.

$${\text{Aqueous solution of trehalose/glucose}} \xrightarrow{\makebox[5cm]{{\text{Conc. Hcl}} 90-100 }^{\circ} \text{C}} {\text{Carbon-nanodots (CNDs)}}$$

### Quantification of the sugars functionalized on the surface of CNDs

The amount of sugar on the nanoparticle surface was quantified using the Anthrone test^28^. Briefly, the CNDs solution was mixed with acidified anthrone solution (80% H_2_SO_4_; w/v) and further heated at 80 °C for 10 min. The solution turned bluish-green due to the formation of a furfural complex. Then the solution was cooled for 5 min in ice water and the absorbance was measured at 630 nm. For the quantification of sugar, at first, a calibration graph for the respective sugars was prepared where absorbance was plotted against sugar concentration. Based on the calibration graph reported in Pradhan et al.^27^, the concentration of respective sugar molecules bound on the respective nanoparticle surface was quantified. The reported calibration graph was as follows: Y = 0.02 [trehalose] and Y = 0.007 [glucose] where Y indicates the absorbance of the bluish-green furfural complex measured at 630 nm and the concentration of sugar was measured in µM.

### Characterization of sugar-terminated CNDs

Characterization of the synthesized sugar-terminated CNDs was done through transmission electron microscopy (TEM), atomic force microscopy (AFM), X-ray diffraction analysis (XRD), zeta potential, and dynamic light scattering (DLS). The optical properties of colloidal CNDs were analyzed by spectrophotometer. Fluorescence emission of CNDs film was imaged using Leica DM3000. The surface functionality of CNDs was analyzed by FTIR.

### Plant material and experimental design

*Vigna radiata* seeds were washed thoroughly with distilled water and sterilized with a 2% sodium hypochlorite solution. The seeds were then sprayed over the cotton bed upon 12 different Petri plates. Three major groups containing four Petri plates were treated with three different concentrations of NaCl (0, 50, and 100 mM). Four Petri plates of each group were treated with distilled water (control), 20 µg/mL CNPT, 20 µg/mL CNPG, and 20 µg/mL trehalose (TRE, molecular control). All germinated seeds were continuously irrigated with 10 mL of each combined treatment (NaCl + dH_2_O/CNDs/TRE) for 7 days. Results were compared by considering 0, 50, and 100 mM NaCl-stressed seedlings without the nanoparticles as the control for their respective sets. All the biological experiments were performed in triplicates and the study methods were designed in a way to comply with the local and national guidelines.

### Germination traits

The germination parameters were assessed by noting down the number of total germinated seeds every day at regular intervals up to 4 days of seedling growth and the calculations were performed as described by Alsaeedi et al.^29^.

$${\text{Germination Percentage }} ({{\text{GP}}}) = \frac{{\text{Total number of seeds germinated}}}{{\text{Total seeds planted}}} \times 100$$

$${\text{Germination energy }} ( {{\text{GE}}} ) = \frac{{{\text{Total number of seeds germinated on }}4{{{\text{th}}}} {\text{ day}}}}{{\text{Total seeds planted}}} \times 100$$

$${\text{Germination speed }} ( {{\text{GS}}}) = \sum \frac{{{\text{Number of seeds germinated at n}}{{{\text{th}}}} {\text{ day}}}}{{\text{Germination day}}}{ }$$

$${\text{Vigour Index }} ( {{\text{VI}}} ) = \frac{{{\text{Dry mass of seedling }} ( {\text{g}}) \times {\text{GP}}}}{100}$$

### Shoot and root length

The length of ten random shoots and roots from each treatment set was noted down and their mean value was considered as a real value for the shoot and root length.

$${\text{Shoot}}/{\text{Root length }} ( {{\text{cm}}}) = \frac{{{\text{Lengths of 10 random seedling shoots}}/{\text{roots}}}}{10}$$

### Estimation of sodium and potassium ions

For the extraction of sodium and potassium ions, 0.2 g of seedlings from each treatment set were homogenized in dH_2_O followed by filtration according to the method of Faiyue et al.^30^. Finally, the ion content was estimated using a flame photometer calibrated with standard solutions of NaCl and KCl.

### Relative water content (RWC)

Relative water content was determined according to the method given by Barr and Weatherly^31^. According to this method, the seedling weights (fresh weight, turgid weight—after soaking the seedlings to full turgidity for 4 h, and dry weight—after removing the total moisture from seedlings) from each treatment set were recorded. RWC was calculated using the following formula:

$${\text{RWC }} ( {{\% }}) = \frac{{{\text{Fresh weight}} - {\text{Dry weight}}}}{{{\text{Turgid weight}} - {\text{Dry weight}}}} \times 100$$

### Photosynthetic pigments

Photosynthetic pigment contents were extracted according to the method of Harborne^32^. According to this method, seedling leaves were homogenized in 80% acetone and filtered through Whatman no. 1 filter paper. The absorbance of the filtrate was taken at 663, 645 and 470 nm to calculate the photosynthetic pigment contents following the formula of Arnon^33^ and Lichtenthaler^34^.

$${\text{Total chlorophyll}} = \left( {20.2 \times {\text{A}}_{645} + 8.02 \times {\text{A}}_{663} } \right)\,\upmu{\text{g mL}}^{ - 1} { }$$

$${\text{Chlorophyll }}a = \left( {12.7 \times {\text{A}}_{663} - 2.69 \times {\text{A}}_{645} } \right)\,\upmu {\text{g mL}}^{ - 1}$$

$${\text{Chlorophyll }}b = \left( {22.9 \times {\text{A}}_{645} - 4.68 \times {\text{A}}_{663} } \right)\,\upmu{\text{g mL}}^{ - 1}$$

$${\text{Total Carotenoid}} = \frac{{\left\{ {\left( {1000 \times {\text{A}}_{470} } \right) - \left( {1.82 \times {\text{Chl}}_{{\text{a}}} } \right) - \left( {85.02 \times {\text{Chl}}_{{\text{b}}} } \right)} \right\}}}{198}\upmu{\text{g mL}}^{ - 1}$$

### Quantification of osmolyte contents

Total and reducing sugars were extracted according to the method of Harborne^32^. For extraction, the plant samples were homogenized in 95% ethanol and boiled in a water bath and finally dissolved in dH_2_O followed by centrifugation. Estimation of total sugar was done using Anthrone reagent according to the standard method of Plummer^28^. Reducing sugar was estimated using alkaline copper tartarate solution and Nelson’s Arsenomolybdate reagent according to the method of Nelson-Somogyi method as described by Plummer^28^. Proline content was determined according to the method of Bates et al.^35^, where the seedlings were homogenized in 10 mL of 3% sulfosalicylic acid and filtered through the Whatman no. 1 filter paper. Proline content from the filtrate was estimated using the Ninhydrin reaction followed by a fractionation step with toluene. Glycine betaine content was determined according to the method of Grieve and Grattans^36^. Extraction was done by homogenizing dried seedlings in 20 mL of dH_2_O followed by constant stirring for 24 h at room temperature. After the completion of stirring, the homogenate was filtered and the glycine betaine content of the filtrate was estimated using 2 N H_2_SO_4,_ potassium tri-iodide solution, and 1,2-dichloroethane.

### Determination of enzymatic antioxidant activities

Superoxide dismutase (SOD) and glutathione reductase (GR) enzymes were extracted by homogenizing the seedlings in 100 mM potassium phosphate buffer (pH 7.6) using PVP under ice-cold conditions. Peroxidase (POX), catalase (CAT), and ascorbate peroxidase (APX) was extracted similarly using 50 mM sodium phosphate buffer of pH 6.8 and 7.2 respectively. The homogenized extracts were then centrifuged at 10,000 rpm for 15 min at 4 °C. The supernatants were considered crude enzyme extracts.

SOD activity was determined following the method of Dhindsa et al.^37^ which is mainly based on the inhibition of the photochemical reduction of NBT at 560 nm. GR activity was determined following the method of Lee and Lee^38^ where enzyme activity was determined by measuring the NADPH oxidation at 340 nm (€ = 6.2 mmol^−1^ cm^−1^). APX activity was determined following the method of Asada and Takahashi^39^, where oxidation of ascorbate (€ = 2.8 mmol^−1^ cm^−1^) continuously decreased the absorbance at 290 nm. POX activity was determined following the method of Chakraborty et al.^40^, where enzyme activity was measured at 460 nm by monitoring the oxidation of o-dianisidine (€ = 11.3 mmol^−1^ cm^−1^) in the presence of H_2_O_2_. CAT activity was determined following the method of Chance and Maehly^41^, where enzyme activity was determined by estimating the decomposition of H_2_O_2_ (€ = 43.6 mol^−1^ cm^−1^) at 240 nm.

### Quantification of non-enzymatic antioxidants

Total phenol along with ortho-dihydroxy phenol was extracted following the method of Mahadevan and Sridhar^42^, by boiling and homogenizing the plant sample with 80% alcohol in dark. Total phenol content was determined following the method of Bray and Thorpe^43^, using Folin ciocalteu’s reagent and Na_2_CO_3_ solution. Ortho-dihydroxy phenol content was determined by the method of Arnow^44^, using Arnow’s reagent along with NaOH solution. Ascorbic acid content was determined following the method of Mukherjee and Choudhuri^45^, where seedlings from each treatment set were homogenized in 6% trichloroacetic acid and filtered. Estimation of ascorbic acid was done using 2% dinitro phenylhydrazine, 10% thiourea, and 80% H_2_SO_4_.

### Estimation of ROS accumulation

H_2_O_2_ was extracted by crushing the plant samples in 10 mL of phosphate buffer (50 mM, pH 6.5) in dark followed by centrifugation at 6000 rpm for 25 min^46^. Estimation of H_2_O_2_ was done using titanium sulphate solution in H_2_SO_4_ followed by centrifugation at 6000 rpm for 15 min. H_2_O_2_ content was calculated using the extinction coefficient value of 0.28 μmol^−1^ cm^−1^. O_2_^**·**−^ was extracted by homogenizing the plant samples in 10 mM phosphate buffer (pH 7) containing 10 mM sodium azide and 0.05% NBT^47^. For estimation, the homogenate was heated in a water bath for 15 min at 85 °C and the absorbance was measured at 580 nm.

### Toxicity analysis of the sugar-terminated CNDs

*Allium cepa* tubers were procured and their old roots and dried shells were cut aside. Then the tubers were allowed to grow for 24 h over the pieces of cotton soaked with distilled water, 20 µg/mL CNPT, 20 µg/mL CNPG, and 20 µg/mL trehalose solutions separately. After 24 h the emerging roots of the tubers were collected for further analysis. The roots were pretreated with paradichlorobenzene and aesculin, fixed with acetoalcohol (1:1 v/v), and stained with acetoorcein^48^. After staining the roots were squashed over a slide with the help of a coverslip and observed under a light microscope (MIKO MD-52-A) at 100 × magnification. Toxicity was assessed by studying mitotic index, root number per tuber, and root length.

### Confocal microscopy of leaves

Section of fixed plant samples was observed under a confocal microscope (ZEISS LSM 880) at 60 × magnification. The excitation wavelength was 488 nm to view the uptake of CNDs into the mesophyll cells treated with CNPT and CNPG solutions. After excitation, the emission was captured at 430–550 nm (green) and 552–630 nm (red) and the merged emission of both was also taken.

## Results and discussion

Salinity is one of the most impactful environmental stresses which generally imparts severe damage to plants. The main effect of salinity stress is reflected in the form of growth retardation^49^. Salinity specifically impairs the water status of plants along with an increase in Na^+^ and Cl^−^ ions, thereby leading to an overwhelming accumulation of ROS. These factors in the long run trigger protein denaturation, DNA damage, lipid peroxidation, and enzyme inhibition in plants^50,51^.

The advent of nanotechnology and its application in agricultural sciences have presented a new dimension to the management of environmental stresses in plants. In this connection, several nanoparticles have been reported to mitigate the negative impacts of salinity in plants^52^. In this context, carbon-nanodots (CNDs) have been shown to ameliorate the saline-alkali stress in tomato plants by triggering the antioxidant enzyme system, promoting nutrient uptake, and enhancing the osmotic adjustment^53^. Similarly, multi-walled carbon nanoparticles have also been reported to ameliorate the effects of salinity stress in *Satureja rechingeri* by improving the root and shoot biomass, and enhancing the accumulation of phenolic acids like caffeic acid and rosmarinic acid^54^.

Functionalization or modification of nanoparticles with different bioactive molecules is expected to increase the overall functionality and efficiency of the nanomaterials^55^. For instance, nano chitosan encapsulated nano silicon was reported to augment the osmotic status, photosynthetic apparatus, and antioxidant activity of wheat plants exposed to salinity stress more efficiently than the bare nano silicon and nano chitosan^56^. Similarly, urea-capped hydroxyapatite nanoparticles were also reported to improve the osmotic status, photosynthetic performance, and antioxidant activity along with ROS scavenging in *Helianthus annuus* exposed to salinity^57^.

Sugars play an important role in the maintenance of photosynthesis, cellular organization, and detoxification of ROS by acting as metabolic signals under stressed conditions. Moreover, they are associated with several physiological and biochemical responses under stress, such as harmonizing enzymatic/antioxidant activity, strengthening membrane integrity, and improving water status^58^. In this connection, trehalose has been known to stabilize protein function acting as an osmoprotectant and osmolyte at the same time^10^. Similarly, glucose has been observed to act as an osmoregulator to facilitate water uptake and retention under salinity stress^59^.

Previously, different type of carbon-based nanoparticles has been functionalized with proline, glycine betaine, and putrescine to enhance their ability to mitigate salinity stress in plants^24,25,60^. However, the role of sugar-terminated CNDs in the alleviation of salinity stress has not been evaluated to date. In this context, the present study has attempted to analyze the efficiency of trehalose and glucose-terminated CNDs (CNPT and CNPG) for the alleviation of salinity stress taking *Vigna radiata* as a model plant*.*

### Attributes of trehalose and glucose-terminated CNDs

Trehalose and glucose-terminated CNDs (CNPT and CNPG) were prepared from molecular trehalose and glucose where the acidified sugar solutions were heated at 95–100 °C leading to complete carbonization of the mixture. It has already been reported that the carbonized core of the nanodot can be encapsulated with a polymeric shell of sugar on the surface resulting in the formation of nanodots terminated with the respective sugar molecule^27^. As the specific CNDs have been prepared from the respective sugar solutions through a low-temperature carbonization process involving condensation-polymerization-dehydration reactions, it was attributed that the corresponding nanodots were terminated with the respective sugar molecules.

**Figure 1:**
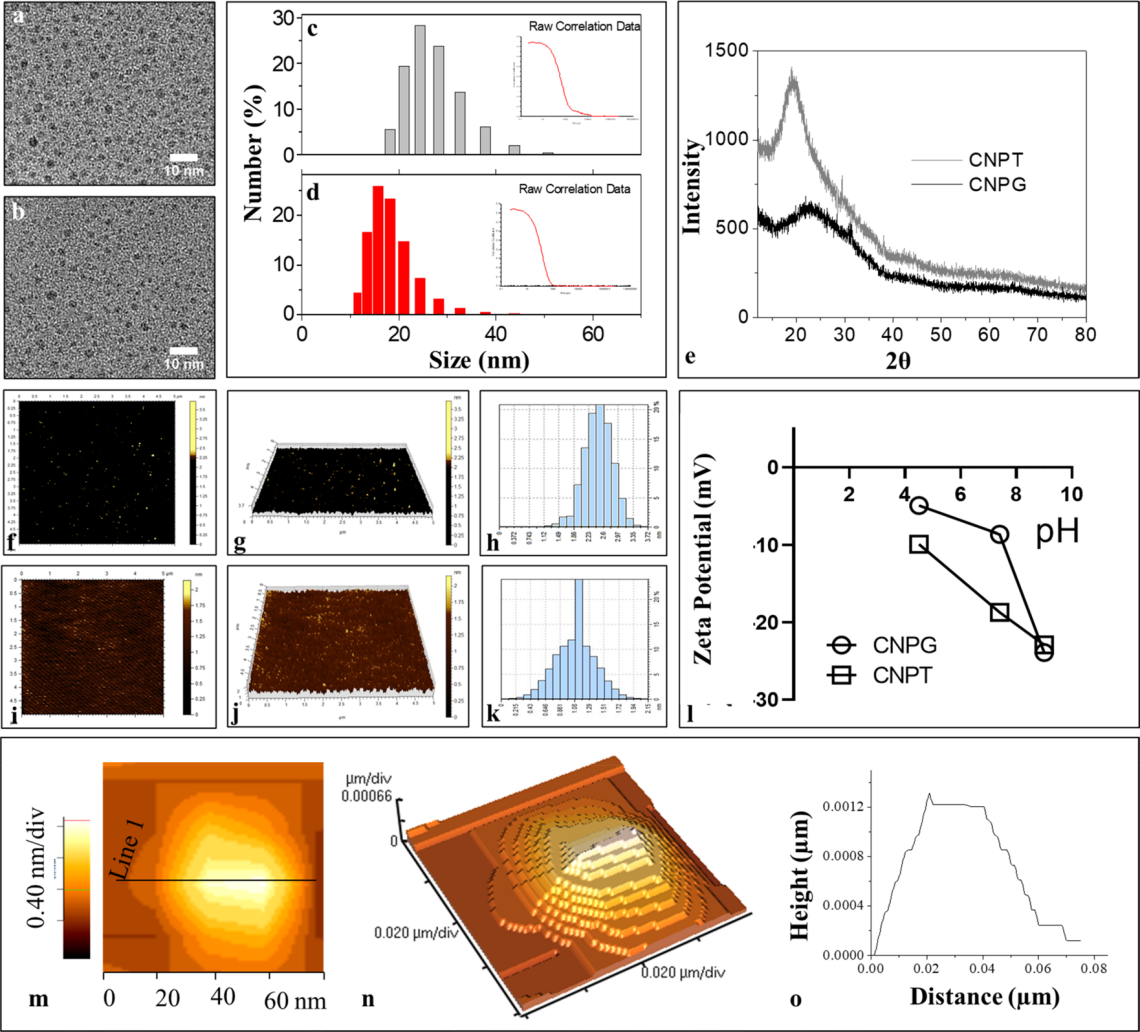
Structural characterization of the carbon-nanodots. (**a**) TEM image of CNP-trehalose (CNPT), (**b**) TEM image of CNP-glucose (CNPG), (**c**) DLS showing the hydrodynamic size of CNPT, (**d**) DLS showing the hydrodynamic size of CNPG, (**e**) XRD spectra of CNPT and CNPG showing broad peak centred at 2θ ~ 20°, (**f**) AFM topography of CNPT, (**g**) Three-dimensional view of CNPT, (**h**) Height profile of CNPT, (**i**) AFM topography of CNPG, (**j**) Three-dimensional view of CNPG, (**k**) Height profile of CNPG, (**l**) Zeta potential of CNPT and CNPG at different pH, (**m**–**o**) Magnified AFM topography of CNPG confirming the plate-like surface morphology of CNDs.

TEM images (Fig. 1a,b) revealed the presence of an inorganic core of CNPT and CNPG with a spherical shape, the size of which ranged from 5 to 10 nm. It has been observed from the dynamic light scattering data, that the colloidal solution of CNPT and CNPG exhibited a hydrodynamic size in the range of 20–40 nm which included the inorganic core of 5–10 nm and polymeric shell of 15–30 nm (Fig. 1c,d). The height of the nanoparticles was imaged using AFM (Fig. 1e–j). The AFM analysis has shown that the average height of the CNDs was within 1–2 nm. In addition, a magnified AFM image of CNPG (Fig. 1m), three-dimensional view (Fig. 1n), and height profile (Fig. 1o) indicated its surface morphology. It confirmed that the CNDs have a plate-like shape with a 5–10 nm core size, 1–2 nm height along with a soft polymeric shell around it which contributes to the 20–40 nm hydrodynamic size of the CNDs. The XRD pattern of CNPT and CNPG (Fig. 1e) have shown a broad peak of 2θ ~ 20° which indicated the highly amorphous nature of the carbon core of both the CNDs synthesized via low-temperature carbonization method. The zeta potential of CNPG varied between − 4 to − 22 mV and − 10 to − 22 mV for CNPT (Fig. 1l) with an increase in the pH of the solution from pH 4.5–9. This indicated the predominant presence of –OH, –COOH like surface functionalities resulting from dehydration-condensation-polymerization of sugars during low-temperature carbonization of CNDs.

**Figure 2:**
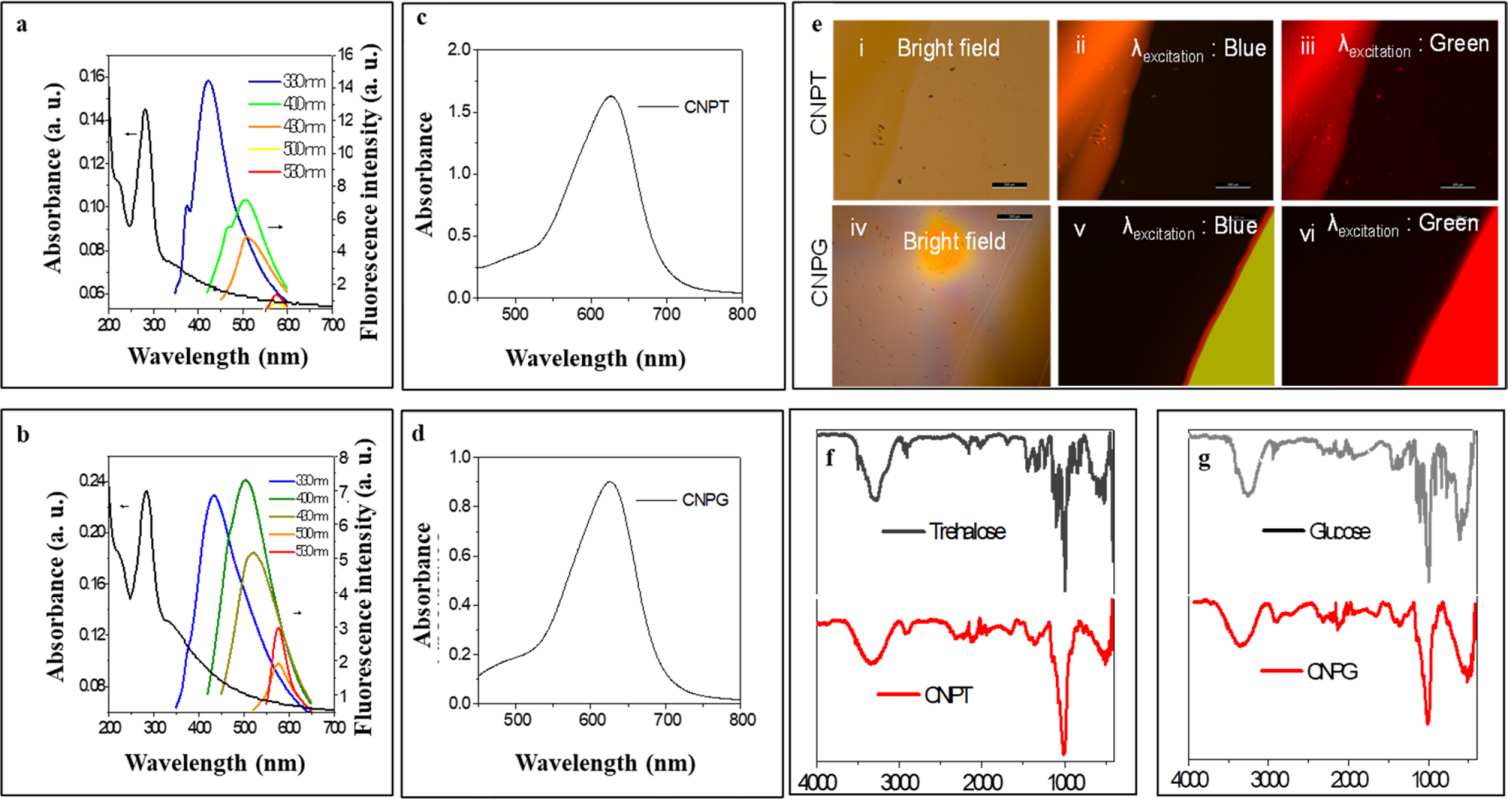
Photophysical properties and surface functionality of carbon-nanodots. (**a**) UV–Vis and fluorescence property of colloidal CNP-trehalose (CNPT), (**b**) UV–Vis and fluorescence property of colloidal CNP-glucose (CNPG); (**c**) Absorbance spectra of CNPT-furfural complex, (**d**) Absorbance spectra of CNPG-furfural complex, (**e**) Emission of CNPT and CNPG film deposited on the glass (i,iv—bright field image, ii,v—emission under blue excitation, iii,vi—emission under green excitation), (**f**) FTIR spectra of CNPT vs molecular trehalose, (**g**) FTIR spectra of CNPG vs molecular glucose.

Further, the CNPT and CNPG exhibited excitation-dependent emission properties (Fig. 2a,b) where emission maxima of both the nanodots shifted from 450 to 600 nm when the excitation wavelength was shifted from 330 to 530 nm. Also, the UV–Visible spectra of CNPT and CNPG (Fig. 2a,b) have shown a broad absorption band in the region of 250–350 nm with maximum absorption at 290 nm which arises due to n–π* (C=O) transition and π–π* (C=C) transition respectively. The brown film of CNPT and CNPG (Fig. 2e (i–vi)) deposited on glass shows respectively orange and greenish-yellow emissions under blue excitation. FTIR analysis of the CNPT and CNPG further proved the presence of functional groups corresponding to the molecular sugar along with the nanodots (Fig. 2f,g). Briefly, the vibrational stretching bands of hydroxyl groups at 3100–3500 cm^−1^, C-H stretching bands at 3000 cm^−1^ and C=O stretching bands at 1720–1640 cm^−1^ were observed for both CNPT and CNPG, which exhibited similarity of the functional groups with the respective sugar molecules. Further, the anthrone test has been used for the quantification of sugars bound to the surface of CNDs. The representative absorbance spectra of CNPT and CNPG (Fig. 2c,d) showed a strong absorbance in 620–630 nm after reaction with a hot acidified anthrone reagent. It indicated that both the CNDs are anthrone positive and using the respective calibration graphs of glucose and trehalose, the amount of sugar molecules on the surface of the respective nanoparticle surface was estimated to comprise 8–10% (w/w) of the nanoparticle. Finally, based on all the analyses it could be concluded that the resultant nanodots (CNPT and CNPG) had 20–40 nm hydrodynamic size (DLS) constituting the 5–10 nm amorphous graphite-like carbon core (TEM) and 15–30 nm soft polymeric shell around it.

### Sugar-terminated CNDs improved the germination and post-germination traits under salinity stress

**Figure 3:**
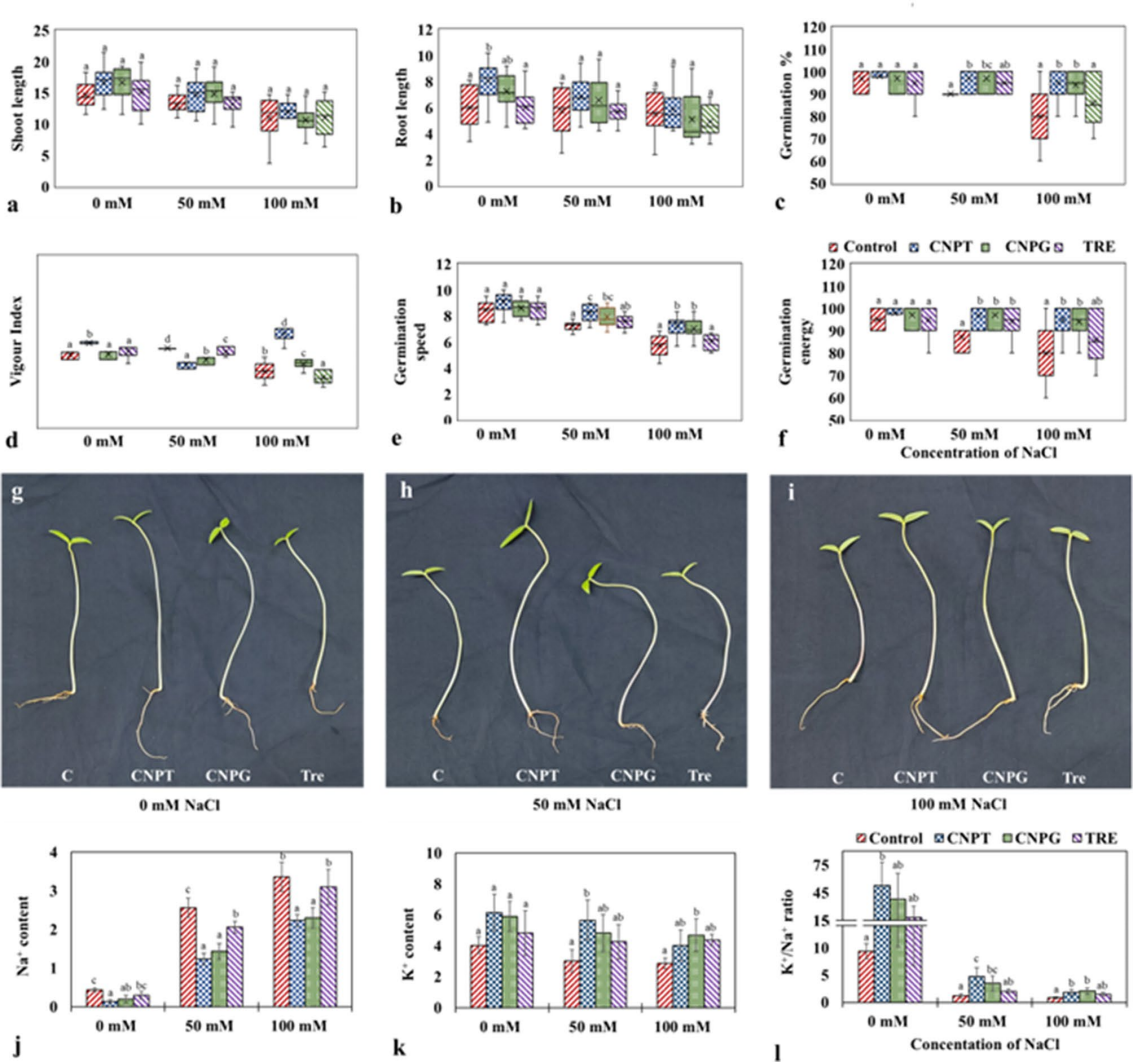
Changes in morphology, germination traits and ionic status of NaCl stressed *Vigna radiata* seedlings following the application of CNPT, CNPG and TRE. (**a**) Shoot length (cm); (**b**) Root length (cm); (**c**) Germination percentage; (**d**) Vigour index; (**e**) Germination speed (Seeds day^-1^); (**f**) Germination energy (%); (**g**–**i**) Images of seedlings after treatment with dH_2_O, CNPT, CNPG, and Trehalose under 0, 50, and 100 mM NaCl stress conditions; (**j**) Sodium content (mg g^−1^ f.w.t); (**k**) Potassium content (mg g^−1^ f.w.t); (**l**) Potassium/Sodium ratio.

The shoot and root length of the seedlings were improved upon the application of CNPT and CNPG both under stressed (+ NaCl) as well as unstressed (− NaCl) conditions (Fig. 3). Though the changes in shoot length were not significant when compared to the respective control sets, it was evident that the application of both CNPT and CNPG augmented the shoot length under the effect of NaCl stress. On the contrary, the root length was found to increase significantly with the application of CNPT under unstressed conditions (− NaCl), however, the increase was not significant when the seedlings were treated with NaCl when compared to the respective controls (+ NaCl). Previous studies have also concluded similar improvements in the shoot and root length of *Satureja rechingeri* plants grown under NaCl and being treated with carbon nanotube at the same time^54^. A different study has also emphasized that the combined application of carbon nanotubes and sodium nitroprusside imparted beneficial effects by increasing the shoot and root length of barley plants exposed to salinity and drought stress^61^. This presents startling evidence that the application of CNDs in combination with other bioactive molecules can augment the overall efficiency of the nanomaterials.

Germination percentage (GP), germination speed (GS), and germination energy (GE) were also observed to increase significantly upon application with CNPT and CNPG when compared to the control set and trehalose treated (used as a control set of molecular sugar) seedlings both under the absence and presence of NaCl (Fig. 3). Vigour index (VI) was also observed to increase significantly in seedlings exposed to 100 mM NaCl upon treatment with CNPT and CNPG, however, when the seedlings were treated with 50 mM NaCl VI was found to decrease with the treatment of both CNPT and CNPG (Fig. 3). These also corroborated with the growth of the germinating seedlings as shown in Fig. 3. Here, CNPT and CNPG demonstrated a better growth of the seedlings both under stressed (+ NaCl) and unstressed (− NaCl) conditions. In this context, previous studies have also described the efficiency of the carbon nanotubes/carbon-based nanoparticles in improving the germination traits of plants grown under saline conditions^62,63^. Pretreatment of seeds with water-soluble carbon nanotubes has also been known to improve the germination parameters of six salt-sensitive varieties of *Lactuca sativa*^21^. Similarly, CNDs have also been observed to increase the germination status of salinity-stressed peanut plants^64^. In agreement with the previous observations, the application of CNPT has shown greater efficiency to improve the germination traits viz*.* GP, VI, GS, and GE (an increase of 1.18, 1.67, 1.26, and 1.16 folds respectively compared to the respective NaCl control set) when treated with 100 mM NaCl. This is in agreement with the previous findings where it has been shown that the functionalization of carbon nanoparticles makes it more efficient in stimulating the breakdown of seed dormancy, thereby, improving the germination rate of plants^65^.

### Sugar-terminated CNDs maintain the ionic balance under salinity stress

Ionic stress is imparted by the overaccumulation of Na^+^ in plant tissues exposed to salinity. Moreover, Na^+^ reprimands the homeostasis of K^+^ in the cytosol that is involved in various biochemical processes. Therefore, the K^+^/Na^+^ ratio appears to be the key factor determining the responses associated with salinity stress tolerance^66^. In this connection, the role of multi-walled carbon nanotubes has been previously reported to support the K^+^/Na^+^ ratio in saline-stressed *Brassica napus*^67^. In agreement with these findings, in the present study also, a significant decrease in Na^+^ content and an increase in K^+^ content were observed along with the application of CNDs (Fig. 3). This resulted in an improved K^+^/Na^+^ ratio in the NaCl-treated seedlings especially when they were simultaneously treated with CNDs (Fig. 3). In this connection, the highest K^+^/Na^+^ ratio was observed with CNPT application among the 50 mM NaCl treated seedlings. On the other hand, the highest K^+^/Na^+^ ratio was observed with CNPG application among the 100 mM NaCl treated seedlings. This improvement in the K^+^/Na^+^ ratio results due to the removal of excessive Na^+^ from the cytosol and the increased uptake of K^+^ at the same time. This phenomenon may involve the SOS1 (salt overly sensitive-1) triggered Na^+^ efflux, NHX (Na^+^/H^+^ exchanger) mediated Na^+^ compartmentation into vacuoles, and KT1 (K^+^ transporter 1) mediated K^+^ influx^67^. Therefore, osmolyte accumulation and Na^+^ compartmentation appear to be the major strategies to overcome salt-induced stress in plants^68^.

### Sugar-terminated CNDs improved the physiological traits under salinity stress

**Figure 4:**
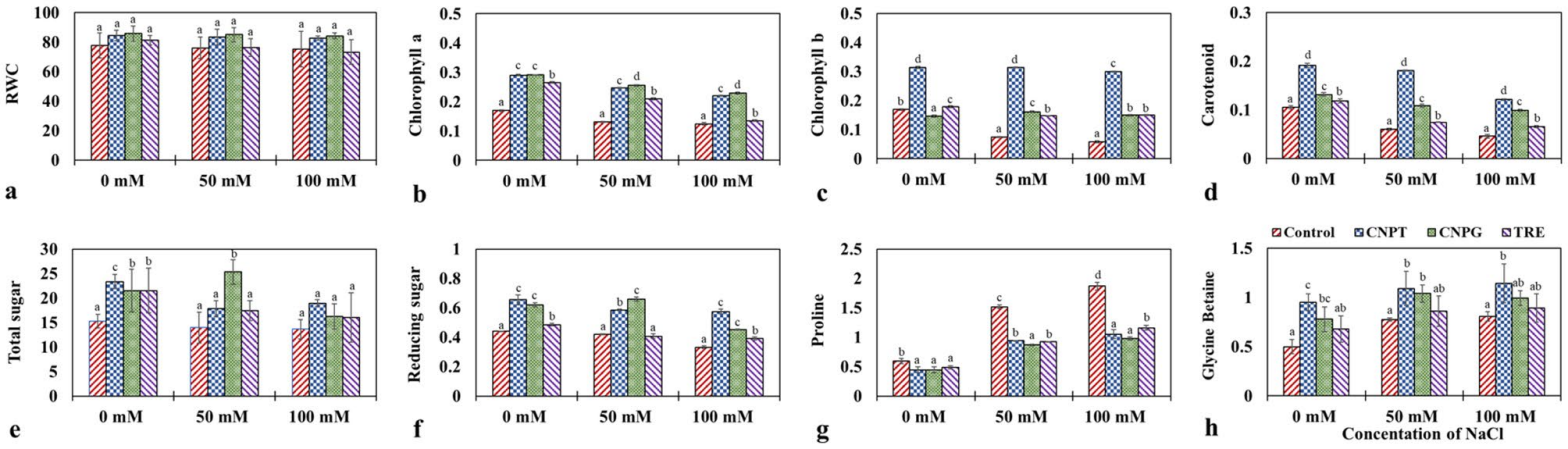
Changes in physiological and osmolyte status of NaCl stressed *Vigna radiata* seedlings following the application of CNPT, CNPG and TRE. (**a**) RWC (%); (**b**) Chlorophyll-*a* (mg g^−1^ f.w.t); (**c**) Chlorophyll-*b* (mg g^−1^ f.w.t); (**d**) Carotenoid (mg g^−1^ f.w.t); (**e**) Total sugar (mg g^−1^ f.w.t); (**f**) Reducing sugar (mg g^−1^ f.w.t); (**g**) Proline (mg g^−1^ f.w.t); (**h**) Glycine betaine (mg g^−1^ d.w.t). *f.w.t* fresh weight tissue, *d.w.t* dry weight tissue.

Relative water content (RWC) provides an idea regarding the water status of plants^69^. Improved RWC is directly associated with the improvement of seed germination traits. In this connection, nitrogen-doped functional CNDs have been known to promote seed germination and plant growth by facilitating the enhanced uptake of water and nutrients^70^. In the present study, RWC was found to reduce in the seedlings upon exposure to increasing NaCl concentrations. Both CNPT and CNPG were observed to increase the RWC under both stressed (+ NaCl) and unstressed (− NaCl) conditions, although the magnitude of increase was not significant (Fig. 4a). In this connection, the highest RWC has been observed in the CNPG treated seedlings followed by CNPT across all the treatment sets (− NaCl and + NaCl). This increase in RWC was approximately 1.11-fold higher than the respective control sets in the case of both 50 and 100 mM NaCl treatment sets. Our findings thus correlate with the previous study by Karami and Sepehri^65^, where the RWC of barley seedlings have been found to improve with the application of carbon nanotubes under both salinity and drought stress. Moreover, the improvement of RWC in saline-stressed plants following the application of CNDs may be explained by the ability of carbon-based nanomaterials in improving the hydraulic conductivity and expression of aquaporins in roots^71^.

Chlorophylls are associated with the key metabolic pathway of photosynthesis and therefore, the status of the same defines the status of plant health. Therefore, a reduction in chlorophyll content often has been used to indicate the susceptibility of plants under stress^72,73^. In the present study, a gradual increase in the concentration of NaCl resulted in the reduction of both chlorophyll-*a* and chlorophyll-*b* content. On the other hand, the application of CNPT and CNPG significantly increased the content of chlorophyll-*a* in the seedlings exposed to NaCl when compared to their respective controls (Fig. 4b). Similarly, a significant increase has been observed in the case of chlorophyll-*b* content when NaCl treated seedlings were applied with CNPT and CNPG, as well as TRE (Fig. 4c). Interestingly, the highest amount of chlorophyll-*a* and *b* were found with the application of CNPG and CNPT respectively, under both stressed (+ NaCl) and unstressed (− NaCl) conditions. CNPG increased the chlorophyll-*a* by about 1.72, 1.94, and 1.84 folds; and CNPT increased the chlorophyll-*b* by about 1.85, 4.19, and 5.16 folds in the case of 0, 50 and 100 mM NaCl treatment sets respectively when compared to their respective controls. A similar observation was found in the case of carotenoid content where CNPT treatment increased its content by about 1.81, 2.99, and 2.64 folds under the effect of 0 mM, 50 mM and 100 mM NaCl respectively when compared to their respective controls (Fig. 4d).

In the present study, the reduction of chlorophyll and carotenoid content of mung bean seedlings exposed to NaCl indicated the negative impact of NaCl on plant growth. However, with the application of CNPT and CNPG the status of the photosynthetic pigments improved under both stressed (+ NaCl) and unstressed (− NaCl) conditions when compared to the respective controls as well as with the TRE treatment sets. In this connection, the role of carbon nanotubes functionalized with the carboxylic acid group has been previously reported to improve the chlorophyll and carotenoid content of *Ocimum basilicum* plants subjected to salinity stress^74^. Moreover, proline functionalized graphene oxide nanoparticles were also reported to restore the biosynthesis of photosynthetic pigments in *Dracocephalum moldavica* under salinity stress^75^. Therefore, the functionalization of carbon nanotubes with beneficial compounds appears to augment the potential of these nanomaterials in maintaining pigment biosynthesis under stressed conditions.

### Sugar-terminated CNDs facilitate osmolyte accumulation under salinity stress

The osmolytes function to counterbalance the imbalance in osmotic pressure under salinity stress^76^. Soluble sugars are well known to augment the osmotic balance of plant cells under salinity stress while acting as an osmolyte. It also promotes the biosynthesis of proline and function of antioxidant defense enzymes which imparts stress ameliorating effects^77^. In the present study, seedlings treated with CNDs showed a higher accumulation of total and reducing sugars when compared to their respective control sets (Fig. 4e,f). At 50 mM NaCl, the highest increase in total sugar content was observed when the seedlings were treated with CNPG (1.81-fold) followed by CNPT (1.3-fold) and TRE (1.2-fold). While at 100 mM NaCl, the CNPT-treated seedlings showed the highest increase of 1.4-fold in total sugar content followed by CNPG and TRE. Similarly, at 50 mM NaCl treatment, the highest increase in reducing sugar content was observed when the seedlings were treated with CNPG (1.57-fold) followed by CNPT (1.4-fold). On the other hand, at 100 mM NaCl treatment, CNPT showed the highest increase in reducing sugar content (1.73-fold) followed by CNPG (1.36-fold) and TRE (1.18-fold). This is in agreement with the findings of Hu et al.^78^ who have observed that the application of carbon nanotubes can enhance the accumulation of carbohydrates (sugar and starch) in *Zea mays* exhibiting their ability to regulate osmotic balance along with their role in carbon and nitrogen assimilation. Therefore, from our findings, it could be concluded that both CNPT and CNPG effectively increased the accumulation of total soluble sugars and reducing sugars under both stressed (+ NaCl) and unstressed (− NaCl) conditions.

Proline is an important osmolyte that generally accumulates in plants in response to salinity stress and helps combat ROS generation^79,80^. The application of carbon nanomaterials has been found to improve the accumulation of proline under salinity stress. For instance, the application of putrescine-functionalized carbon quantum dots to grapevines under salinity stress has been observed to release the negative effects of salt stress by increasing the accumulation of proline^25^. Similarly, proline functionalized graphene oxide nanoparticles were found to increase the biosynthesis of proline in *Dracocephalum moldavica* under salinity stress^75^. However, in the present study, proline accumulation was generally observed to increase along with the increasing concentrations of NaCl but not with the application of CNDs. The increase in the proline content of the seedlings exposed to NaCl was, therefore, found to be relieved by the application of CNPT, CNPG, and trehalose (Fig. 4g). About 1.77 and 1.91-fold decrease in the accumulation of proline was observed with the application of CNPT and CNPG respectively at 100 mM NaCl which was also lower than that of trehalose-treated seedlings. This reduction in the accumulation of proline content of seedlings treated with CNPT, CNPG, and TRE under salinity stress exhibited lesser dependency on proline biosynthesis for stress tolerance.

Glycine betaine is a quaternary ammonium compound—a compatible solute that has various key functions in cellular osmotic adjustment, improving water-use efficiency and maintaining cell organelles^81^. Glycine betaine has also been known to impart a protective role in plants exposed to salinity stress^82–84^. In our study, glycine betaine content was observed to increase along with the increasing concentrations of NaCl. The results show an additive increment in glycine betaine content (CNPT > CNPG > trehalose) in all the seedlings under stressed (+ NaCl) as well as unstressed (− NaCl) conditions when compared to their respective controls (Fig. 4h). A significant increase in glycine betaine content (1.41 and 1.76-fold) was observed in CNPT treated seedlings under both 50 and 100 mM NaCl treatment which was also greater than that of the trehalose treated seedlings. From this result, it is clear that the CNDs are more capable than the molecular trehalose in the alleviation of salinity stress. This may be attributed to the CNDs mediated enhancement in the accumulation of sugar molecules inside the plant tissues. In this connection, it can also be realized that the accumulation of sugars can enhance the rate of ATP synthesis via Kreb’s cycle which in turn supports the energy-dependent biosynthesis of CDP-choline—an important precursor of glycine betaine^85,86^. Therefore, the increased accumulation of reducing sugar and glycine betaine content as a result of CNPT/CNPG application plays a major role in the alleviation of stress, relieving the proline biosynthesis pathway, thus supporting the physiological status of the seedlings exposed to salinity^87^.

### Sugar-terminated CNDs regulate the activity of antioxidants under salinity stress

Enzymatic and non-enzymatic antioxidants play a vital role in the scavenging of excess ROS generated as a result of salinity stress. The major enzymatic antioxidants which are usually studied includes superoxide dismutase (SOD), ascorbate peroxidase (APX), glutathione reductase (GR), catalase (CAT), and peroxidase (POX). Among these, SOD is mainly associated with the detoxification of superoxide radicals, whereas the other enzymes are involved in negating the deleterious effects of H_2_O_2_^88^. Among the non-enzymatic antioxidants—ascorbate, carotenoids, and phenolic compounds are well known to mitigate the oxidative damage caused by ROS^89^.

**Figure 5:**
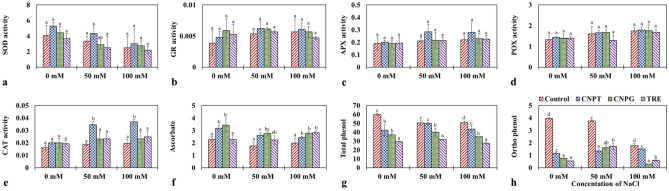
Changes in enzymatic and non-enzymatic antioxidant activities of NaCl stressed *Vigna radiata* seedlings following the application of CNPT, CNPG and TRE. (**a**) Superoxide dismutase (Enzyme units mg protein^−1^); (**b**) Glutathione reductase (μmol NADPH mg protein^−1^ min^−1^); (**c**) Ascorbate peroxidase (mmol ascorbate mg protein^−1^ min^−1^); (**d**) Peroxidase (mmol o-dianisidine mg protein^−1^ min^−1^); (**e**) Catalase (mol H_2_O_2_ mg protein^−1^ min^−1^); (**f**) Ascorbic acid (mg g^−1^ f.w.t); (**g**) Total phenol (mg g^−1^ f.w.t); (**h**) Orthodihydroxy phenol (mg g^−1^ f.w.t).

In the present study, a decrease in the activity of SOD was generally observed along with an increase in the concentration of NaCl. However, in the seedlings treated with 50 mM NaCl, CNPT imparted a significantly enhanced activity of SOD compared to the control set. Moreover, the CNPT-treated seedlings showed higher SOD activity followed by CNPG and TRE in the case of both stressed (+ NaCl) and unstressed (− NaCl) conditions (Fig. 5a). GR activity was observed to increase gradually with the increasing concentrations of NaCl. However, the highest activity of GR was also observed in the case of CNPT-treated seedlings, which exhibited an increase of 1.16 and 1.08-fold at 50 and 100 mM NaCl treatments respectively when compared to the control set (Fig. 5b). A similar trend was observed for APX activity, where the highest activity was again observed in the seedlings treated with CNPT under both the concentrations of NaCl. In this regard, about 1.34 and 1.28-fold increase in APX activity was observed at 50 and 100 mM NaCl treatments respectively (Fig. 5c). POX activity was also found to gradually increase with the increasing concentrations of NaCl. Although the values did not differ significantly, the results distinctively showed higher activity of POX in both CNPT and CNPG-treated seedlings (Fig. 5d). CNPT also imparted a significant increase in CAT activity subjected to 50 and 100 mM NaCl treatments, which was about 1.9-fold greater when compared to the respective control sets. An increase in CAT activity was also observed with CNPG and TRE treatment, but the increase was not significant when compared to their respective controls (Fig. 5e).

These results revealed that the activity of all the enzymatic antioxidants increased with the increasing concentrations of NaCl except for SOD. Application of CNPT and CNPG further increased the antioxidant activities across all the treatments when compared to their respective control sets (+ NaCl/− NaCl) (Fig. 5a-e). These results are in agreement with the findings of Shekhawat et al.^90^ who also found a similar increase in the activities of SOD, CAT, POX, and APX in saline-stressed *Vigna radiata* plants while treated with carbon nanoparticles. Another study also revealed that the treatment of grape seedlings with multiwalled carbon nanotubes under salinity stress imparted an enhanced activity of several enzymes viz*.* SOD, POX, and CAT^61^. Moreover, the modification of carbon nanotubes with carboxylic acid as a functional group effectively enhanced the activities of APX and CAT, which was shown to contribute towards the augmentation of stress tolerance in saline-stressed *Ocimum basilicum*^74^.

In the present study, among the non-enzymatic antioxidants - the ascorbate content was found to decrease by 1.29 and 1.14-fold subjected to 50 mM and 100 mM NaCl stress respectively. However, the CNPT and CNPG treatments enhanced the ascorbate content in seedlings at both concentrations of NaCl (Fig. 5f). For instance, at 50 mM NaCl, the increase in ascorbate content was found to be about 1.48 and 1.56-fold in the seedlings treated with CNPT and CNPG respectively. However, at 100 mM NaCl, TRE treatment showed the highest ascorbate content followed by CNPG and CNPT when compared to the control set. On the other hand, total phenol and ortho-dihydroxy phenol were observed to decrease when treated with CNPT, CNPG, and trehalose. The present study indicated that increasing concentrations of NaCl generally decreased the phenol content of seedlings. The lower accumulation of phenols was observed with the application of CNPT and CNPG irrespective of the NaCl concentrations. However, the lowest accumulation of phenols was observed in the case of TRE-treated seedlings (Fig. 5g). Similar results have been observed in the case of ortho dihydroxy phenol content. Under 100 mM NaCl, the CNPT-treated seedlings exhibited a minimal decrease (1.17-fold), while the CNPG and TRE-treated seedlings showed 6.09 and 2.96-fold lower accumulation of ortho-dihydroxy phenol respectively in comparison to the control set (Fig. 5h).

The findings of the present study are in agreement with the results of López-Vargas et al.^91^ who emphasized that the carbon nanomaterials can induce the accumulation of phenols and ascorbate that can play a vital role in the improvement of tolerance in saline-stressed tomato seedlings. Similarly, other studies have also presented the efficacy of functionalized carbon nanomaterials in improving the stress tolerance of plants. In this connection, the phenol content was observed to increase under saline stressed plants when treated with glycine betaine-functionalized graphene oxide nanoparticles^24,92^. However, in our study, phenol content has been found to reduce with the applications of CNDs, which may be attributed to the superior contribution of enzymatic antioxidants in conferring stress tolerance.

### Sugar-terminated CNDs alleviate ROS accumulation under salinity stress

**Figure 6:**
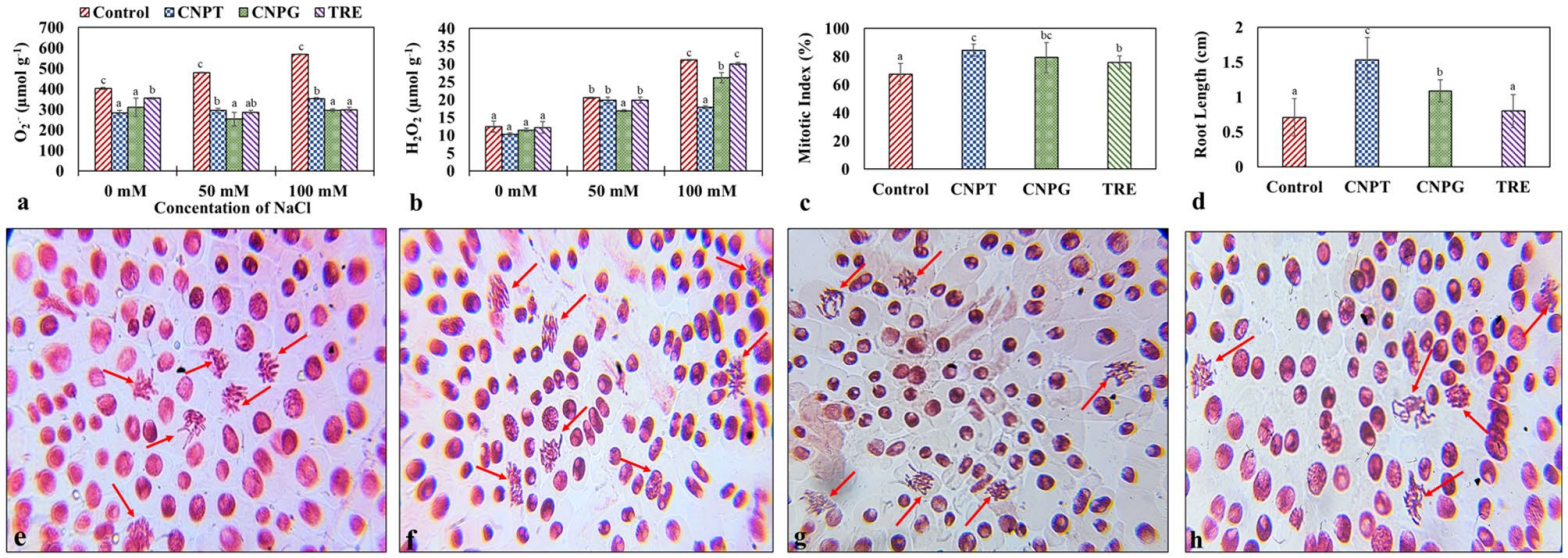
Changes in ROS accumulation of NaCl stressed *Vigna radiata* seedlings following the application of CNPT, CNPG and TRE, and *Allium cepa* root tip bioassay. (**a**) O_2_^**·**−^ content (μmol g^−1^ f.w.t), and (**b**) H_2_O_2_ content (μmol g^−1^ f.w.t) of saline stressed *Vigna radiata* seedlings; (**c**) Mitotic index (%) of *Allium cepa* root tip; (**d**) Root length of *Allium cepa*; (**e**-**h**) Microscopic cellular images of *Allium cepa* root tip treated with dH_2_O, CNPT, CNPG, and TRE solutions respectively. Red arrows (**e**–**h**) point out the healthy dividing cells.

Increased accumulation of osmolytes not only provides osmoprotection to plants but also stimulates antioxidant activities under stress conditions^93^. Moreover, the osmolytes can scavenge the toxic ROS directly and thus improve the antioxidant defense system of plants by triggering the expression of defense-related genes^94,95^. In this purview, the scavenging of reactive oxygen species (ROS) in plants is modulated by the increased accumulation of osmolytes, antioxidant activities, and expression of stress-responsive genes^96^. In our study, both superoxide anion (O_2_^**·**−^) and hydrogen peroxide (H_2_O_2_) content were generally observed to increase with the increasing concentrations of NaCl. However, with the application of CNPT, CNPG, and TRE the accumulation of ROS reduced under stressed conditions (+ NaCl) (Fig. 6a,b). Comparatively, CNPT and CNPG were more efficient in scavenging the excess ROS, which can be explained by the efficiency of these nanomaterials in improving the osmolyte accumulation, that directly or indirectly triggered the antioxidant defense system of the seedlings under NaCl stress. For instance, in the case of seedlings treated with 50 mM NaCl, application with CNPG resulted in the lowest accumulation of O_2_^**·**−^ (about a 1.9-fold decrease than the control set) followed by TRE (1.67-fold). However, in the case of 100 mM NaCl-treated seedlings, CNPT exhibited the highest reduction in H_2_O_2_ content (1.74-fold) followed by CNPG (1.19-fold). Previous studies conducted in *Ocimum basilicum* have also exhibited a similar reduction in ROS accumulation when treated with glycine betaine functionalized graphene oxide nanoparticles^24^. Similarly, Gohari et al.^60^ have also shown that the proline functionalized carbon-nanodots efficiently scavenged the excess ROS accumulated in grapevine plants under salinity stress.

### Cytotoxicity and genotoxicity analysis of sugar-terminated CNDs

The application of nanotechnology for improving agricultural productivity has been grabbing increased attention than ever before. However, studies reporting the toxicity of the engineered nanoparticles have been limited. The toxicity assessment of biogenic silver nanoparticles on *Allium cepa* root tip and flowering bud implied that even a low dose of the nanoparticles can induce significant clastogenic effects on both meristematic and reproductive cells^97^. The toxicity effect of silver nanoparticles was also assessed upon roots of *Pisum sativum*, *Drimia indica*, and *Triticum aestivum*^98–100^. Except for these, toxicity assessments of other nanoparticles viz. silica, titanium dioxide, aluminium, zinc, gold, copper, iron, and magnesium using the *Allium cepa* root cell division bioassay has been evaluated^101–106^. Also, the toxicity of nitrogen-doped carbon dots and bare carbon dots in the form of inhibitory effects on primary root growth of *Arabidopsis thaliana* have been reported^107,108^. In the present study, the toxicity analysis of the sugar-terminated CNDs was assessed by studying the genotoxicity and cytotoxicity using *Allium cepa* root tip bioassay. The root tip bioassay has revealed that the CNDs exhibited no trace of toxicity (chromosomal aberration), instead, the nanomaterials conferred beneficial attributes in terms of improved cell division and a simultaneous increase in root length (Fig. 6c-h). The highest mitotic index was found in the case of CNPT-treated onion bulbs (84.63%) followed by CNPG (79.37%), and TRE (75.91%) (Fig. 6c). Similar results were observed in terms of root length where the highest increase in root length was observed in the case of CNPT-treated bulbs (1.53 cm) followed by CNPG (1.09 cm), and TRE (0.8 cm) (Fig. 6d). In conclusion, the presence of no chromosomal aberration (Fig. 6e-h), improved mitotic index, and root length with the treatment of CNPT and CNPG proved its harmless nature as well as confirmed its beneficial effects on cell growth.

### Sugar mediated stress tolerance is dependent on the uptake of sugar-terminated CNDs

**Figure 7:**
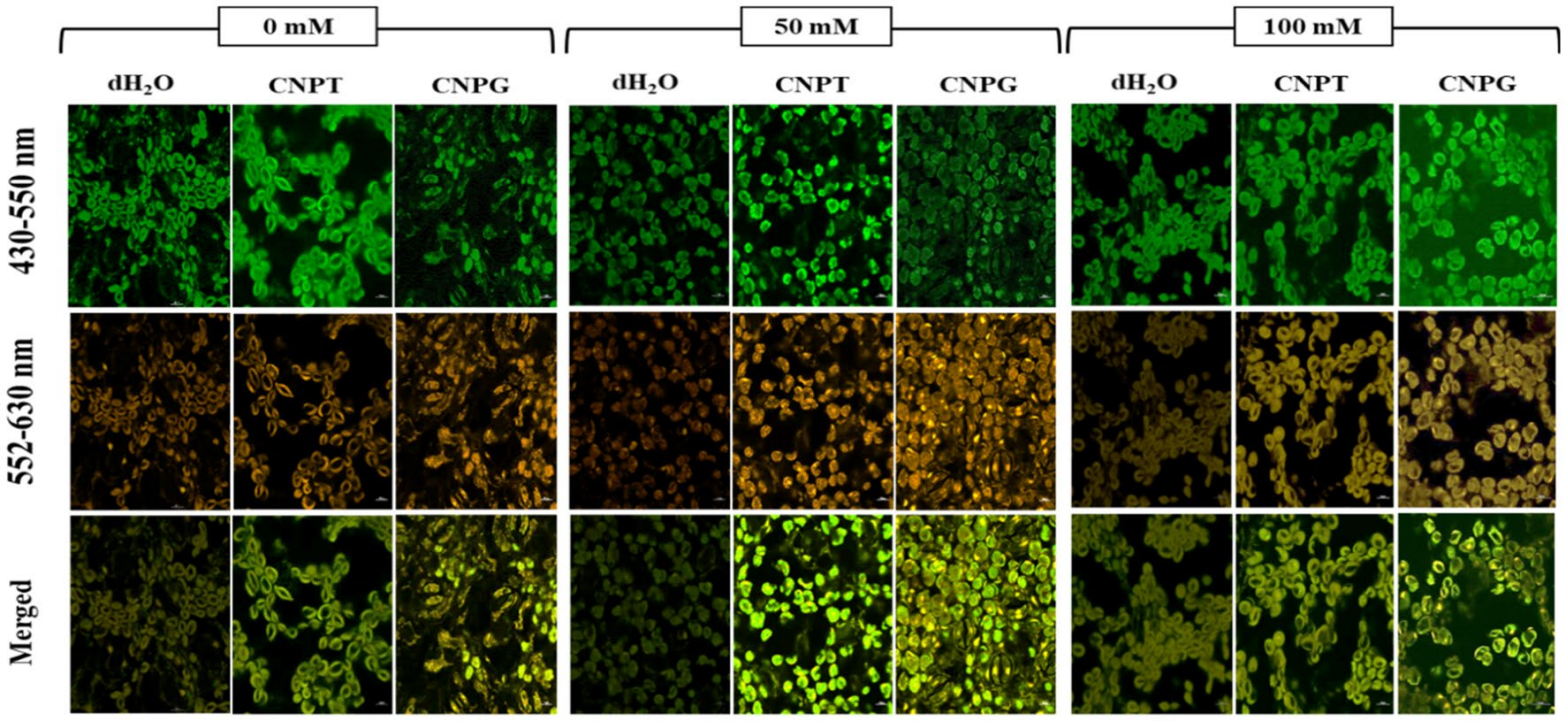
Confocal microscopy images (60 ×) showing the uptake of carbon-nanodots into the mesophyll cells treated with CNPT and CNPG solution. Excitation was done at 488 nm and emission was captured at 430–550 nm (Green—showing the fluorescence of mesophyll cells) and 552–630 nm (Red—showing the fluorescence of uptaken CNDs). The merged images also show the fluorescence of CNDs.

In the present study, confocal microscopy was used to analyse the uptake of CNDs. In this connection, it has been found that under green spectrum emission (430–550 nm), the fluorescence of mesophyll tissues was highest in CNPT-treated seedlings followed by CNPG treatment across all the treatment sets viz. 0, 50, and 100 mM NaCl. These findings also corroborated the results of photosynthetic pigments, where the pigment content was observed to increase with CNPT and CNPG treatment under both stressed (+ NaCl) and unstressed (− NaCl) conditions. Under red spectrum emission (552–630 nm) fluorescence of the uptaken CNDs was highest in CNPT-treated seedlings and moderate in CNPG-treated seedlings subjected to NaCl treatments, thus, indicating the better uptake of CNPT than CNPG into the seedlings. The highest uptake of CNPT was also confirmed by the merged picture of confocal microscopy where better fluorescence was observed in the case of CNPT-treated leaves followed by CNPG-treated leaves (Fig. 7).

The better uptake of CNDs in the present study can also be correlated with the negative zeta potential of the nanomaterials. In this connection, it has been previously shown that the COOH-functionalized carbon nanotubes with higher negative surface charge resulted in reduced aggregation of the nanoparticles, thereby, making them more water soluble and easily uptaken by plant roots^109^. Moreover, the uptake of functionalized nanoparticles depends upon one or more specific cellular stimuli viz*.* pH, redox potential changes, enzymatic activation, and thermal gradients^110^. The release of functionalized properties inside the cell from the CNDs surface mainly depends upon the cellular pH. Zare et al.^111^ briefly discussed the mechanism of controlled and targeted release of functionalized molecules from various kinds of CNDs, where the pH was proved to be the main stimulus. In this connection, the present study has also shown that the increase in pH resulted in increasing the negative zeta potential of both CNPT and CNPG. More specifically, at physiological pH, the zeta potential in the range of − 8 to − 18 mV can be useful for avoiding the aggregation of CNDs and ensuring the precise delivery of the sugar molecules.

## Conclusion

**Figure 8:**
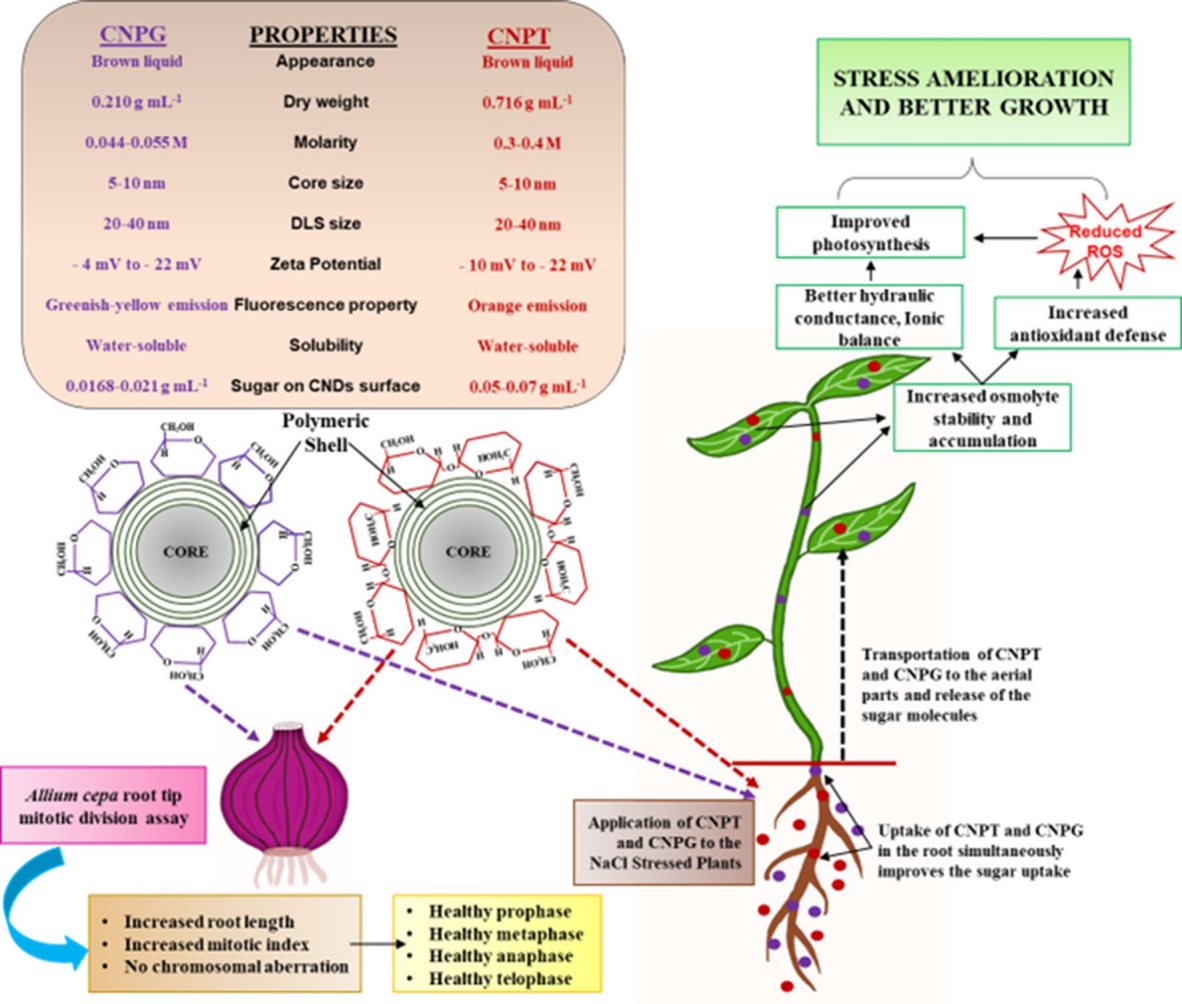
Schematic representation showing the structural and functional attributes of the CNDs and the brief mechanism associated with the alleviation of NaCl stress in *Vigna radiata*. The application of both CNPT and CNPG reduced the negative impacts of salinity stress by modulating the physiological and biochemical parameters subjected to salinity stress. Briefly, the CNDs facilitated the uptake of sugar molecules, thereby, improving the osmotic status along with the improvement in pigment biosynthesis, antioxidant defense, and ion homeostasis. Moreover, CNDs exhibited no toxicity in the *Allium cepa* root tip bioassay, indicating their suitability for plant-based applications.

The present study reveals that the sugar-terminated CNDs alleviate the salinity stress of *Vigna radiata* seedlings more efficiently in comparison to the untreated and molecular trehalose-treated seedlings. Functionalized CNDs were found to be efficiently uptaken by the growing seedlings as evident from the images of confocal microscopy and therefore, facilitated the mobilization of sugar molecules into the aerial parts. Increased uptake of sugars along with CNDs improved the K^+^/Na^+^ ratio by reducing the uptake of Na^+^ and improving the K^+^ accumulation in the seedlings. The CNDs also improved seed germination, growth parameters, photosynthetic pigments, osmotic balance, and most importantly increased antioxidant defense. A mechanistic action of the CNDs (CNPT and CNPG) facilitating the uptake of sugar molecules that helped in the alleviation of salinity stress and growth improvement of the seedlings have been presented in Fig. 8 in this connection. Both the CNDs showed excellent potential for the palliation of salinity stress, however, CNPT elicited slightly more effective responses than the CNPG. The toxicity assessment of the sugar-terminated CNDs revealed their non-toxic nature, thereby, upholding their promise for the development of nanofertilizers. Further studies in this connection will enlighten the potential of these unique nanomaterials in the field of sustainable agriculture.

## Data Availability

All data generated or analysed during this study are included in this article.
